# Binaural Localization Development and the Effect of SmartSound iQ with SCAN in Children with Cochlear Implants

**DOI:** 10.3390/audiolres15060163

**Published:** 2025-11-24

**Authors:** Abdulaziz Alasmi, Mada Aljabr, Dalal Alrushaydan, Hassan Yalcouy, Fida Almuhawas

**Affiliations:** 1 King Abdullah Ear Specialist Center (KAESC), King Abdulaziz University Hospital, King Saud University Medical City, Riyadh 12629, Saudi Arabia; fmuhawas@ksu.edu.sa; 2 Cochlear Arabia Regional Headquarters, Riyadh 12361, Saudi Arabia; maljabr@cochlear.com (M.A.); dalrushaydan@cochlear.com (D.A.); hyalcouy@cochlear.com (H.Y.); 3 Department of Otolaryngology–Head and Neck Surgery, College of Medicine, King Saud University, Riyadh 11451, Saudi Arabia

**Keywords:** deafness, hearing aid, sound localization, auditory localization, auditory perception

## Abstract

Background: Binaural hearing is crucial for spatial auditory perception, including sound localization. Cochlear implants (CIs) are commonly used in children with congenital deafness to support binaural auditory development, but the extent to which they facilitate effective localization remains unclear. Objective: This study evaluates the impact of CIs on binaural functional auditory development and sound localization in children with congenital deafness, considering factors such as age at implantation, hearing experience, and CI laterality. Methods: Thirty-eight CI-assisted children (aged 4–9 years) underwent directional hearing assessments using the “Erfassung des Richtungshörens bei Kindern (ERKI)” device. Localization accuracy was analyzed across various noise stimuli, and correlations with audiometric parameters and CI history were examined. Results: Localization accuracy improved with age and CI experience. Bilateral CI users outperformed unilateral users, particularly with pulse pink noise stimuli. The use of SmartSound iQ with SCAN technology enhanced localization, especially in younger children. Conclusion: CIs support binaural functional auditory development in children with congenital deafness, with localization skills improving over time. Bilateral implantation and early intervention may further enhance outcomes, warranting continued research.

## 1. Introduction

The ability to hear enables people to experience sound and receive information, which aids their competency and ultimately survival [1]. The nature of sound, being a form of energy that travels away from its source in a consistent, predictable manner, means that part of the information a sound can provide is its direction of travel and therefore the location of its source [2]. This inherent spatial and directional information can confer both situational and social awareness to the listener that can enhance their physical safety and social competency, and therefore quality of life [3,4].

Sound localization is one of the most fundamental tasks of the human auditory system [5]. Full perception of acoustic space, including directional hearing, requires binaural hearing, or in other words, having two functioning ears; slight differences in the perception of the acoustic properties of a sound between the left and right ear, such as signal intensity and arrival time, can be processed by the brain to calculate source locations [6]. This ability to process binaural auditory information typically develops during the first years of childhood. Nowadays, children with congenital deafness are routinely treated with cochlear implants (CI), with some undergoing surgery in the first year of their life, to coincide with the natural progression of functional binaural auditory development during early childhood. It is established that CI surgery often enables such children to develop binaural hearing and therefore sound localization abilities [6]. This is particularly important for children with single-sided deafness (SSD), who often show marked localization deficits compared with normal-hearing peers [7]. A recent comprehensive review confirmed that CI in SSD children can restore localization ability, reduce auditory fatigue, and improve academic performance [8].

Beyond SSD, evidence consistently shows that bilateral implantation provides substantial advantages for spatial hearing. Children with bilateral CIs exhibit significantly greater localization accuracy than those with a single CI, especially in tasks involving horizontal sound placement [9]. These benefits appear to strengthen with age and accumulated CI experience [10], and early bilateral implantation during toddlerhood can support the emergence of directional hearing, as shown in “reaching for sound” tasks [11]. Indeed, when bilateral implantation occurs before age five significantly improves localization compared to implantation after age ten [12].

In addition to laterality and surgical gap between implantations, other factors may sharpen localization ability [13]. Several studies highlight the benefits of signal processing technologies, such as automatic scene classification, especially SCAN-enabled feature. While these features have primarily been studied in relation to speech-in-noise perception, others suggest potential advantages in spatial hearing [12,14,15]. Emerging evidence suggests that these benefits may extend to spatial hearing, where sharpening of binaural cues could improve localization [16].

Importantly, the influence of CIs may not be limited to auditory performance. Optimized auditory input via CIs also correlates with improved cognitive outcomes in children, such as better verbal, figural, and arithmetic fluency [15]. Interestingly, in sequential CI users, the time gap between implants does not consistently predict outcomes like speech perception or binaural performance [15]. Nonetheless, bilateral use remains a consistent factor associated with improved localization and overall listening performance [14].

In order to assess the progress of a child’s directional hearing and binaural auditory development, audiologists measure any interaural differences in the child’s sound perception. Advanced diagnostic localization testing is facilitated by an Erfassung des Richtungshorens bei Kindern (ERKI) device which stimulates the child’s frontal horizontal plane with up to 37 distinct sound sources, making it suitable for assessment of both children with normal hearing and those with CI-assisted hearing [16,17,18,19].

In 2010, Salloum et al. compared the interaural level differences (ILD) and localization abilities of primary school children with normal and CI-assisted hearing [18]. They found that the CI-assisted children were less able to localized sounds than those with normal hearing, and suggested that these differences might be due to receiving the first implant late, or due to a longer duration between the first and second implant [17]. This study, however, did not set out to determine whether impaired localization development in CI-assisted children improves over time or not.

Similarly, Grieco-Calub and Litovsky (2010) found that children with bilateral CIs (BiCIs) demonstrated superior localization performance compared to their unilaterally implanted peers, with notable gains observed in tasks assessing horizontal sound placement [9].

Later, in 2015, Zheng et al. further investigated the development of binaural hearing and sound localization in a group of CI-assisted children aged 4 to 9 years old, who had from 1 to 4 years of bilateral experience [1]. A very different localization pattern was revealed, although the results did not inform on whether earlier implantation was necessary, or if the children’s ability to effectively localize sound would improve over time [1].

To enhance pediatric audiology practices and deepen our understanding of how CIs influence auditory development in congenitally deaf children, we investigated binaural hearing and sound localization abilities in young CI users. Our study was guided by the following hypotheses: 1. the age of the child plays a significant role in auditory development; 2. greater hearing experience, particularly with earlier implantation, leads to better outcomes; and 3. specific sound processing features, such as automatic scene classification (e.g., SCAN), may improve localization by sharpening spatial auditory cues.

## 2. Materials and Methods

This pediatric audiology study was designed to investigate the development of binaural hearing and auditory localization abilities in children who underwent cochlear implantation. Pediatric patients aged between 4 and 9 years underwent directional hearing assessments using an ERKI device to evaluate their binaural hearing and localization competency.

### 2.1. Inclusion and Exclusion Criteria

All patients with congenital deafness who underwent CI surgery at a tertiary CI center in the last 9 years and were aged between 4 and 9 years at the time of selection were offered an assessment of their binaural hearing and localization ability. Children who could not complete the assessments due to behavioral, cognitive, or developmental reasons were excluded. Parental consent for each child and attendance at their routine CI follow-up appointments were also required for admission into the study.

### 2.2. Data Collection

Upon enrolment, the following information was collected: age at directional hearing assessment, age of first surgery, age of second surgery, duration of fitting with hearing aids prior to CI, hearing experience after CI, details of CI or behind-the-ear device, sex, first language, hearing loss reason (if there is one), aided hearing test results, and speech audiometry results.

### 2.3. Assessments

Directional hearing assessments were performed directly after each child’s regular CI follow-up appointment, which includes the following routine pediatric audiology assessments: tympanometry, free field aided test audiometry (with CI using narrow band bursts from loudspeaker), and free field speech audiometry, as well as the regular CI-fitting procedure.

The study directional hearing assessments were conducted using an ERKI device. Directional hearing assessments were conducted using the ERKI (Erfassung des Richtungshörens bei Kindern) system, version AT1000, manufactured by HörTech GmbH, Oldenburg, Germany. The child faced directly forward while hidden speakers emitted a wide range of acoustic stimuli towards them from any direction within a 180° circumference (Supplementary Figure S2). After each acoustic stimuli, the child was able to move a steering device to indicate in which direction they had localized the sound to have come from. A light-emitting diode (LED) device that moved in concordance with the steering movement provided visual feedback during the localization process. Due to the young age of the children, qualified and experienced audiologists judged the reliability of the children’s answers. The accuracy of each child’s localization ability was determined and recorded as a percentage.

The ERKI assessment protocol is based on an auditory signal at 65 dB SPL with 90°± in both directions and a 10° angle resolution, which is divided into 5 blocks, each with a different stimulus type. The first four blocks covered stimulus types of white noise, pink noise, speech noise, and pulse pink noise, respectively, all assessed with the assistance of SmartSound iQ with SCAN technology. Then fifth block was white noise SCAN off. All children enrolled in this study underwent training with a full test prior to the commencement of the protocol. ERKI results in this study are expressed as the percentage of correct directional responses based on the child’s indicated sound source, rather than angular RMS error (in degrees), which is commonly used in adult or lab-based localization studies. Percentage-based scoring is more behaviorally accessible for younger populations and reflects how often a child correctly identifies or closely approximates the direction of the stimulus. Higher ERKI percentages indicate better localization performance. Each test block includes multiple trials, and the average percentage of correct responses is calculated to derive the final score.

SmartSound iQ with SCAN technology is designed to replicate natural hearing by automatically adapting captured sound to adjust for the influence of the surrounding auditory environment.

Captured sound is analyzed to identify and extract specific signal features, which assists the SCAN automatic scene classifier in analyzing the surrounding acoustics to determine the most probable listening environment from a closed set of scenes (Speech in Noise, Speech, Noise, Wind, Quiet, and Music). The identified scene automatically informs SmartSound iQ on optimization of the sound to provide improved hearing.

### 2.4. Statistical Analyses

Data were entered into Microsoft Excel and then imported for analysis using IBM SPSS Statistics v.25. Categorical variables are described using frequencies and percentages. Quantitative variables were assessed for normality using Shapiro–Wilk. Normally distributed data are summarized using means and standard deviations (SD). Non-normally distributed data are summarized using medians and interquartile ranges. Paired-samples t-tests were used to compare outcomes between conditions within the same group (e.g., sound in noise with SCAN on vs. SCAN off). Fisher’s exact test was used for comparisons of categorical variables when expected cell counts were below the recommended thresholds. Pearson correlation coefficients were used to assess the association between normally distributed continuous variables. Spearman’s rank correlation coefficients were used for non-normally distributed continuous variables. Independent-samples t-tests were used to compare continuous variables between two independent groups with normally distributed data. The Mann–Whitney U test was used for comparisons between two independent groups with non-normally distributed data. One-way analysis of variance (ANOVA) was used for comparisons of normally distributed data across more than two groups. The Kruskal–Wallis H test was used for comparisons of non-normally distributed data across more than two groups. Post hoc comparisons for significant results were performed using Bonferroni correction for multiple comparisons. A significance level of α = 0.05 was used throughout the analyses.

### 2.5. Ethical Approval

The study was approved by the Institutional Review Board (IRB) Approval of Research Project No. E-22-6784.

## 3. Results

### 3.1. Patients

A total of 57 patients were identified as meeting the enrolment criteria (Supplementary Figure S1). Of these, 9 were excluded: 3 were determined to be non-users, 3 had reported behavioral issues, 2 had developmental/global delays, and 1 was reported to have cognitive issues. Subsequently, 48 CI-assisted children were contacted, of whom 3 did not respond, 6 refused to visit the clinic for testing and 1 was not in the country, resulting in 38 being enrolled into the study.

Table 1 summarizes the baseline characteristics of the study participants (n = 38). The majority of participants were male (62.5%) with ages ranging from 4 to 9 years. Laterality of CIs was predominantly bilateral (64.6%). CP1000 was the most common type of implant used (62.5%), followed by CP910 (16.7%). Regarding stimulation parameters, most patients used default settings (41.7%), while nearly 40% (38.9%) used PW37. Congenital hearing loss was the primary etiology for CI (97.2%). Type A tympanometry was observed in the majority of patients (76.3%). The mean age of hearing experience from the first surgery was 3.75 years (SD = 1.95 years). Among bilateral implant users, the mean age of hearing experience from the second surgery was 3.29 years (SD = 1.97 years), with an average time difference of 258.13 days (SD = 450.59 days) between the two surgeries. Pre-operative hearing aid use averaged 7.64 months (SD = 7.69 months).

### 3.2. Binaural Hearing and Sound Localization

The auditory thresholds and ERKI results are shown in Table 2. Aided average thresholds were similar for the right ear (28.26 dB HL; SD: 6.52 dB) and left ear (27.08 dB HL; SD: 5.84 dB). Speech reception thresholds (SRT) were at around 22.9 dB HL in both ears. ERKI results revealed the children performed best with white noise (mean 31.5%, SD: 13.07%), followed by pink noise (25.74%, SD: 13.10%), speech noise (25.39%, SD:17.17%), and pulse pink noise (23.18%, SD: 16.83%). Notably, ERKI scores dropped significantly when the SCAN was turned off during white noise presentation (20.16%, SD: 16.05%).

### 3.3. Reliability of Localization Results

The majority of children demonstrated reliable ERKI results (~60%). The reliability of the children’s results improved with increasing age, with the difference between the youngest (4–5 years old) and the oldest (8–9 years old) age groups being statistically significant (*p* = 0.044).

### 3.4. Sound Localization with/Without SCAN

**Figure 1:**
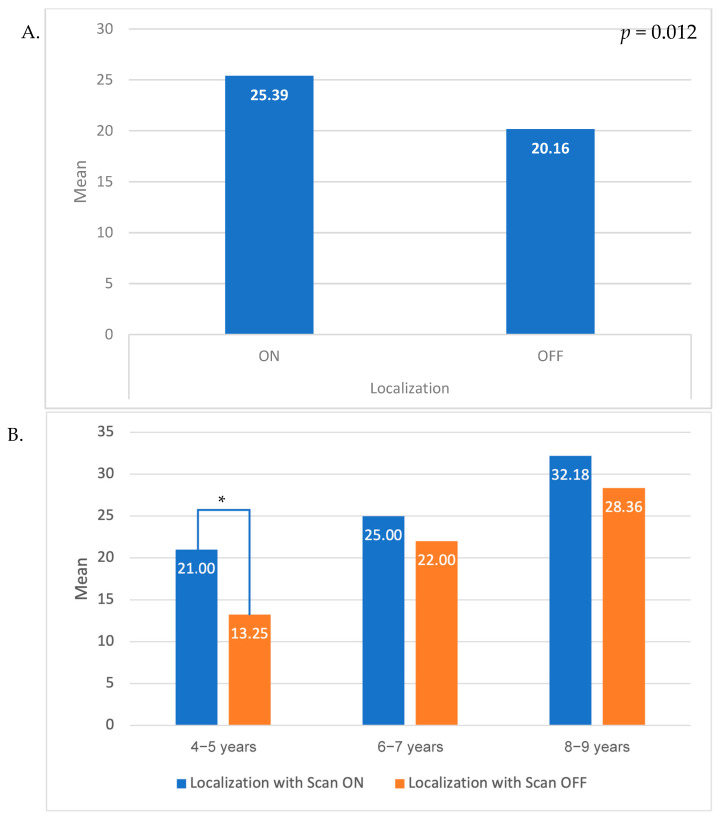
Difference in localization results with scan ON/OFF (**A**). In the whole sample; (**B**). Stratified by age groups). An asterisk (*) indicates a statistically significant difference between Scan ON and Scan OFF conditions (*p* < 0.05).

ERKI localization scores across all ages, with SCAN either on or off, are presented in Figure 1A, with associated scores for individual age groups presented in Figure 1B. Mean localization with white noise score across all ages. White noise was significantly higher with scan ON compared to scan OFF in the whole sample (*p* < 0.001) and in the age group of 4–5 years (*p* < 0.001). The scores appear consistently higher with the scan ON compared to scan OFF across all age groups, even if it did not reach statistical significance in the older age groups.

### 3.5. Correlations Between Audiological Measures

**Figure 2:**
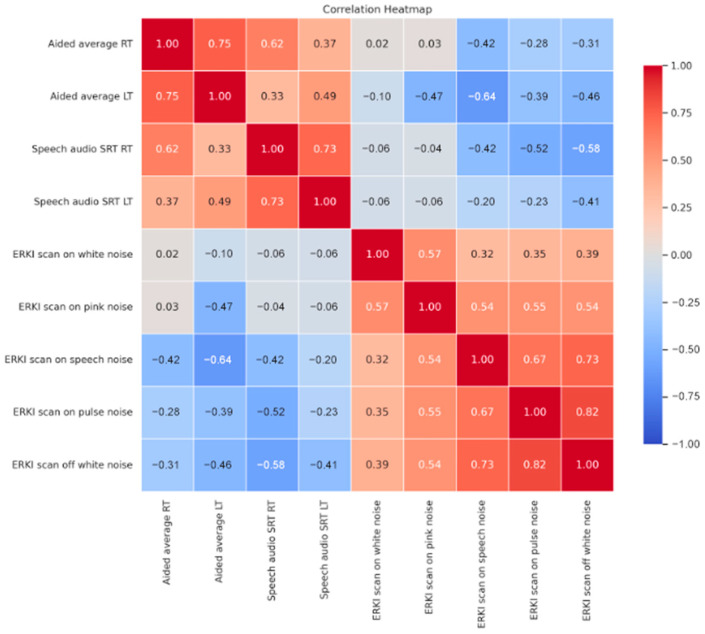
Correlation heatmap of audiological measurements.

Figure 2 presents the correlation coefficients between various audiological measures obtained from the study participants. Aided average thresholds (RT and LT) demonstrated positive correlation (r = 0.618, *p* < 0.001 and r = 0.491, *p* = 0.009) with SRTs RT and LT, respectively. Notably, aided thresholds and SRTs both exhibited significant negative correlations with ERKI scores obtained during white noise presentation, with SCAN on and off, and with ERKI s scores with different stimulus noise types (pink, speech, pulse pink). ERKI scan scores were also all intercorrelated significantly, except for white noise and speech noise with scan ON.

### 3.6. Impact of Age on ERKI Results

To investigate the influence of age on ERKI results, one-way ANOVA was used for normally distributed data and Kruskal–Wallis H test for non-normally distributed data (Supplementary Figure S3). While no significant differences were found for ERKI scores obtained with white noise (*p* = 0.303), pink noise (*p* = 0.153), or speech noise (*p* = 0.350) across age groups, a significant difference was observed for pulse pink noise scores (*p* = 0.046). Post hoc comparisons using Bonferroni correction revealed a significant difference in pulse pink noise scores between the youngest age group (4–5 years old) and the oldest age group (8–9 years old).

Table S1 explores the correlations between different ERKI measurements (SCAN on) stratified by age groups. In the age group of 4–5 years, only one significant correlation was found between speech noise and pulse pink noise (r = 0.583, *p* = 0.018). In older groups, more linear relationships were found.

### 3.7. CI Laterality (Unilateral vs. Bilateral)

Associations between ERKI results and CI laterality are presented in Table 3 No statistically significant differences were found in ERKI scores between unilateral and bilateral CI users for white noise (*p* = 0.371), pink noise (*p* = 0.660), or speech noise with SCAN on (*p* = 0.230). However, scores for both pulse pink noise stimulus (median: 11 dB, IQR: 0–24 dB) and ERKI speech noise during SCAN off (median: 11 dB, IQR: 0–24 dB) were significantly lower in the unilateral CI group compared with the bilateral CI group (median: 29 dB, IQR: 21–37 dB and median: 21 dB, IQR: 16–32 dB; *p* = 0.045 and *p* = 0.038, respectively).

### 3.8. Correlation Between Measurements and Hearing Experience of Unilateral/Bilateral CIs

Table S2 explores the correlations between various measures and hearing experience in children with unilateral or bilateral CIs. In unilateral implant recipients (n = 12), longer hearing experience since the first surgery was associated with better scores on ERKI assessments with white noise SCAN off (r = 0.617, *p* = 0.033). This group also demonstrated a positive correlation between duration of pre-operative hearing aid use and ERKI scores with speech noise (r = 0.753, *p* = 0.007).

In contrast, in bilateral implant users (n = 24), longer hearing experience since both the first and second surgeries was associated with significantly better scores on ERKI results. Further, in this bilateral implant group, there were no significant correlations between the time interval between the two surgeries and ERKI results, nor with pre-operative hearing aid use and ERKI results.

## 4. Discussion

This pediatric audiology investigation was designed to further understanding of the impact of CIs on the development of binaural hearing and sound localization in children. Previous studies compared sound localization abilities of children with normal hearing and those using CIs, and found that CI users typically show reduced localization accuracy, likely due to limitations in the spatial cues provided by CI systems, such as degraded or absent interaural time differences (ITDs), rather than a lack of auditory development per se [1,18,19,20,21,22].

Further, while these earlier studies did demonstrate that CI users do develop sound localization, the impact of early CI implantation, as compared with later implantation, on binaural processing development remains unassessed [23]. Similarly, the impact of the duration between first and second CI surgeries has not been fully quantified [9]. Therefore, this study enrolled patients as young as 4 years of age, with the aim of analyzing the correlations between duration of CI users hearing experience and localization ability. It is important to note that our study does not aim to investigate neural or brain development directly. All findings relate to functional auditory performance as measured by behavioral assessments of sound localization.

The impact of age at the time the CIs were received on binaural hearing development can firstly be seen by the fact that ERKI scores for localization improve with increasing age, with older patients, who had longer CI users hearing experience, having higher scores (Figure 1B). Our observation that localization skills improved with age is consistent with broader auditory development findings reported by Skarzynski [19,24] et al. (2024) and align with the developmental patterns reported by Litovsky (2016), who used a novel “reaching for sound” paradigm in toddlers aged 27–42 months [11,19]. They demonstrated that even at this very young age, bilateral CI users can discriminate sound direction and identify source locations, albeit with lower accuracy than normal-hearing peers [11,19]. Their findings highlight that bilateral CI recipients begin utilizing spatial cues early, and that these skills mature with continued bilateral hearing experience [11,19].

In our study, this age-related trend was more pronounced with the SCAN on, with the oldest group (8–9 years) exhibiting the highest scores. Additionally, the youngest age group (4–5 years) demonstrated the lowest scores under white noise SCAN off. These findings support the conclusion that binaural hearing and localization ability in CI-assisted children improves with increasing hearing experience. This developmental trajectory is consistent with findings from Asp et al. (2022), who demonstrated that infants and young children receiving early bilateral implantation achieved horizontal localization development rates comparable to their normal-hearing peers [20]. However, children of younger age were also less likely to provide reliable test results, so comparisons across ages may not be representative of differences in localization ability alone.

Recent pediatric evidence shows that such automatic scene classification can enhance speech perception in noise (Ching et al., 2024), suggesting potential benefits for localization as well [25]. In our study, localization scores were consistently higher with SCAN on compared to SCAN off across the entire study population, although this difference did not reach statistical significance in the older age groups. This improvement was most evident in children aged 4 and 5 who exhibited significantly lower localization scores with SCAN off, which may be attributed to the extended duration of the assessment and a decline in reliability for this age group as the assessment progressed.

While SCAN processing has the potential to modify access to binaural cues, particularly ILDs and ITDs, depending on microphone directionality and noise suppression algorithms, we hypothesize that in our sample, SCAN may have improved localization by enhancing the clarity of target signals and reducing background interference. This may have supported behavioral attention to spatial cues, particularly in younger children or those with limited hearing experience. This interpretation is consistent with findings from Mauger et al. (2014), who demonstrated that SCAN-enabled processing in the Nucleus 6 processor significantly improved speech recognition in noise via automatic scene classification and adaptive directional microphone activation [26]. Although Mauger et al. did not assess localization performance, their results support the premise that SCAN can optimize access to salient auditory cues in challenging listening environments, a concept our study extends to the domain of pediatric spatial hearing [26].

The improved localization under SCAN ON conditions may be attributed to the activation of automatic input processing features, including standard directionality and ADRO [26]. Standard directionality mildly attenuates rear input, enhancing spatial contrast in the frontal field, while ADRO dynamically optimizes gain across frequencies to maintain audibility [19]. These features may support localization by enhancing the clarity of spatial cues, particularly in young children who depend on salient auditory input for spatial learning [24]. The alignment between SCAN’s signal-enhancement strategy and our testing methodology, which is focused on target signals from ±90°, likely contributed to the observed improvement, as the absence of competing noise allowed SCAN’s enhancement features (e.g., ADRO and standard directionality) to effectively amplify salient spatial cues [24]. We believe this synergy played a key role in the statistically significant results. Additionally, SCAN OFF was administered last in the test protocol, and reduced attention or fatigue may have contributed to lower scores in that condition [27,28].

The ERKI system used in our study is designed to assess sound localization specifically in the horizontal plane, using an array of loudspeakers arranged in a semicircular arc at ear level. As such, our results primarily reflect localization based on interaural level and time difference cues. We acknowledge that the external ear (pinna) plays a critical role in spatial hearing by introducing spectral filtering important for vertical-plane and front, back discrimination in normal-hearing individuals [28]. This dimension of localization is typically underrepresented in cochlear implant users due to microphone placement behind the ear and limited transmission of spectral cues [29].

The use of a pulse pink noise stimulus proved advantageous in hearing tests for children, a finding that was corroborated in the ERKI testing. Pulse pink noise consistently improved ERKI scores among children using CIs, with these improvements becoming more pronounced with increasing age. Additionally, a significant difference in ERKI scores was observed between unilateral and bilateral CI users when pulse pink noise was used. Notably, children appeared to demonstrate greater interest and engagement during localization assessments involving pulse pink noise compared to other types of noise stimuli.

The superior performance of bilateral users for specific stimuli aligns with broader evidence on the spatial hearing benefits of bilateral implantation. For example, Jaisinghani et al. (2024) reported that children who received their second CI by age 5 achieved markedly better localization than those implanted after age 10 [12]. While our study did not detect a significant effect of the interval between surgeries, this may be due to the relatively small number of sequentially implanted participants or differences in age at second implantation compared with the Jaisinghani cohort. Comparable laterality-related benefits were also noted by Astefanei et al. (2025), who found substantial localization improvements in both pediatric and adult recipients of cochlear or bone conduction implants, with the largest gains in bilateral users [14]. Collectively, these findings underscore the importance of both stimulus type and CI laterality in assessing and fostering binaural hearing development in pediatric CI recipients.

Aided thresholds and SRTs both exhibited significant negative correlations with ERKI scores obtained during white noise presentation, with SCAN on and off, and with ERKI scores with different stimulus types (speech, pink, pulse pink noises). This suggests that poorer aided thresholds and SRTs were associated with lower ERKI scores, potentially indicating reduced hearing ability. Aided and speech scores can reflect hearing experience and device usage time. This also showed that better aided and speech scores corelate with better ERKI score. Importantly, improved auditory access, as reflected in better ERKI scores, may also support language and cognitive abilities. Skrbic et al. (2023) reported that children with CIs outperformed peers in verbal, figural, and arithmetic fluency, reinforcing the broader developmental value of optimized hearing [15].

Interestingly, the duration between the first and second surgeries in sequential cases did not appear to influence localization ability in children. This aligns with the findings of Almeida et al. (2019), who reported no statistically significant correlation between inter-implant interval and outcomes such as speech perception or binaural performance in children and adolescents with sequential implants [30]. However, it is important to note that the number of sequential cases included in this study was relatively low, which may limit the generalizability of this observation. As anticipated, younger patients provided less reliable assessment results compared to older patients. Nevertheless, it remains unclear whether this discrepancy was primarily due to their less developed binaural processing abilities or their limited comprehension of the ERKI test relative to older children. It could be argued, however, that communication skills are at least partially dependent on overall hearing ability, potentially contributing to the variability in assessment reliability among younger participants.

## 5. Limitations

The ERKI localization assessment is a new test in pediatric audiology; so, most children enrolled in this study had no prior experience with it. Also lack of a control group or the small sample size for sequential CI cases. A formal power analysis was not conducted prior to study initiation, given the exploratory design and the limited size of the eligible pediatric CI population. While all implanted children meeting the inclusion criteria were contacted, the absence of a predefined sample size calculation should be considered when interpreting the findings. Although SCAN technology is capable of classifying musical environments, our study did not include music stimuli in the localization testing protocol.

### Areas for Further Research

Future studies should investigate the impact of SCAN technology on localization ability in children using cochlear implants, as this area remains underexplored. In particular, examining how SCAN processing influences spatial hearing in response to music stimuli would be valuable, given the role of music in auditory quality of life and real-world listening engagement.

## 6. Conclusions

Localization ability improves with age in children using cochlear implants, particularly with the use of pulse pink noise stimuli. Children aged 4–5 years using cochlear implants may benefit from additional training and shorter test durations to improve the reliability of ERKI localization assessments.

## Figures and Tables

**Table 1:** Baseline characteristics and demographics of the study participants (N = 38).

	N	%
Sex		
Male	23	60.5%
Female	15	39.5%
Age at examination		
4	9	23.7%
5	7	18.4%
6	8	21.1%
7	3	7.9%
8	6	15.8%
9	5	13.2%
Laterality		
Unilateral	12	31.6%
Bilateral	26	68.4%
Type of device		
CP910	4	10.5%
CP950	3	7.9%
CP1000	26	68.4%
CP1150	4	10.5%
CP910 & CP950	1	2.6%
SP_parameters		
PW37	14	38.9%
PW50	2	5.6%
PW75	1	2.8%
Default	15	41.7%
Mixed	4	11.1%
Hearing loss reason		
Congenital	35	97.2%
Not congenital/Tuberous sclerosis	1	2.8%
Tympanometry type		
A	29	76.3%
B	3	7.9%
C	3	7.9%
Occluded	1	2.6%
Mixed	2	5.3%
	Mean	Standard deviation
Hearing experience age from first surgery (in years)	3.75	1.95
Hearing experience age from second surgery (in years) (among bilateral implant patients)	3.29	1.97
Date difference between 1st and 2nd surgeries (in days) (among bilateral implant patients)	239.36	374.00
Hearing aid usage pre-implantation (in months)	7.64	7.69

**Table 2:** Test results of the sample.

		Mean	SD
Aided average RT (dB HL)		28.26	6.52
Aided average LT (dB HL)		27.08	5.84
Speech audio SRT RT (dB HL)		22.97	6.58
Speech audio SRT LT (dB HL)		22.96	8.58
ERKI SCAN on white noise (%)		31.5	13.07
ERKI SCAN on pink noise (%)		25.74	13.1
ERKI SCAN on speech noise (%)		25.39	17.17
ERKI SCAN on pulse pink noise (%)		23.18	16.83
ERKI SCAN off white noise (%)		20.16	16.05
		N	%
Reliability	Good	23	60.50%
	Bad	15	39.50%

**Table 3:** Association between the different measurements and laterality.

		ERKI SCAN on White Noise (%)	ERKI SCAN on Pink Noise (%)	ERKI SCAN on Speech Noise (%)	ERKI SCAN on Pulse Pink Noise (%)	ERKI SCAN off White Noise (%)
	N	Mean (SD)	Mean (SD)	Median (Q1; Q3)	Median (Q1; Q3)	Median (Q1; Q3)
Laterality						
Unilateral	17	29 (10)	24 (14)	21 (0; 35)	11 (0; 24)	11 (0; 24)
Bilateral	31	33 (14)	26 (13)	32 (21; 37)	29 (21; 37)	21 (16; 32)
*p*		0.371	0.660	0.230	0.045	0.038

## Data Availability

The data used and/or analyzed in this study is available and will be provided by the corresponding author upon request.

## Supplementary Materials

The following supporting information can be downloaded at: https://www.mdpi.com/article/10.3390/audiolres15060163/s1, Figure S1: Flow diagram of patient disposition; Figure S2: Set-up of the directional hearing assessment; Figure S3: Differences in ERKI results with SCAN on between the different age groups: A. White noise; B. Pink noise; C. Speech in noise; D. Pulse noise; Table S1: Correlation between ERKI measurements with SCAN on stratified by age group; Table S2: Correlation between measurements and unilateral/bilateral CIs hearing experience.

## Abbreviations

The following abbreviations are used in this manuscript:

CI	Cochlear Implant
ERKI	Erfassung des Richtungshörens bei Kindern (Localization Testing in Children)
ILD	Interaural Level Difference
LED	Light-Emitting Diode
SPL	Sound Pressure Level
SRT	Speech Reception Threshold
RT	Right (ear)
LT	Left (ear)
dB HL	Decibels Hearing Level
APC	Article Processing Charge
SD	Standard Deviation
IQR	Interquartile Range
ANOVA	Analysis of Variance
SPSS	Statistical Package for the Social Sciences
