# Supplementary material for: Binaural Localization Development and the Effect of SmartSound iQ with SCAN in Children with Cochlear Implants
Source: Audiol Res. 2025 Nov 24;15(6):163. doi: 10.3390/audiolres15060163 (PMC12729642; doi:10.3390/audiolres15060163)
Supplement: Supplementary file 1 [file audiolres-15-00163-s001.zip › audiolres-3707565-supplementary.pdf]

[Appendix / Supplementary Materials]

Table S1. Correlation between ERKI measurements with SCAN on stratified by age group

			ERKI SCAN on pink noise (%)	ERKI SCAN on speech noise (%)	ERKI SCAN on pulse pink noise (%)
4-5 years (N=16)	ERKI SCAN on white noise (%)	Correlation Coefficient	-0.051	-0.146	0.076
		p	*0.851*	*0.591*	*0.779*
	ERKI SCAN on pink noise (%)	Correlation Coefficient		0.207	-0.166
		p		*0.441*	*0.539*
	ERKI SCAN on speech noise (%)	Correlation Coefficient			.583*
		p			*0.018*
6-7 years (N=11)	ERKI SCAN on white noise (%)	Correlation Coefficient	.763**	0.598	.621*
		p	*0.006*	*0.052*	*0.041*
	ERKI SCAN on pink noise (%)	Correlation Coefficient		0.456	.697*
		p		*0.158*	*0.017*
	ERKI SCAN on speech noise (%)	Correlation Coefficient			0.567
		p			*0.069*
8-9 years (N=11)	ERKI SCAN on white noise (%)	Correlation Coefficient	.813**	0.601	0.555
		p	*0.002*	*0.05*	*0.076*
	ERKI SCAN on pink noise (%)	Correlation Coefficient		.729*	.817**
		p		*0.011*	*0.002*
	ERKI SCAN on speech noise (%)	Correlation Coefficient			.838**
		p			*0.001*

Table S2. Correlation between measurements and unilateral/bilateral CIs hearing experience

			ERKI SCAN on white noise (%)	ERKI SCAN on pink noise (%)	ERKI SCAN on speech noise (%)	ERKI SCAN on pulse pink noise (%)	ERKI SCAN off speech noise (%)
Unilateral	Hearing experience age from first surgery (years)	Correlation Coefficient	0.617*	0.235	-0.118	0.125	0.004
	N=12	*p*	*0.033*	*0.462*	*0.715*	*0.7*	*0.991*
	Hearing aid usage pre-implantation (months)	Correlation Coefficient	-0.047	0.594	0.753**	0.386	0.595
	N=11	*p*	*0.891*	*0.054*	*0.007*	*0.242*	*0.053*
Bilateral	Hearing experience age from first surgery (years)	Correlation Coefficient	0.301	0.548**	0.477*	0.627**	0.499*
	N=24	*p*	*0.152*	*0.006*	*0.018*	*0.001*	*0.013*
	Hearing experience age from second surgery (years)	Correlation Coefficient	0.423*	0.528**	0.434*	0.640**	0.640**
	N=24	*p*	*0.039*	*0.008*	*0.034*	*0.001*	*0.001*
	Date difference between first and second surgeries (days)	Correlation Coefficient	-0.022	0.21	0.232	0.028	0.036
	N=25	*p*	*0.917*	*0.314*	*0.265*	*0.894*	*0.863*
	Hearing aid usage pre-implantation (months)	Correlation Coefficient	0.097	-0.003	-0.112	-0.145	0.022
	N=25	*p*	*0.644*	*0.987*	*0.594*	*0.488*	*0.918*

*p is <0.05; **p is <0.01,
Results calculated using Spearman test are in red.

Figure S1. Flow diagram of patient disposition

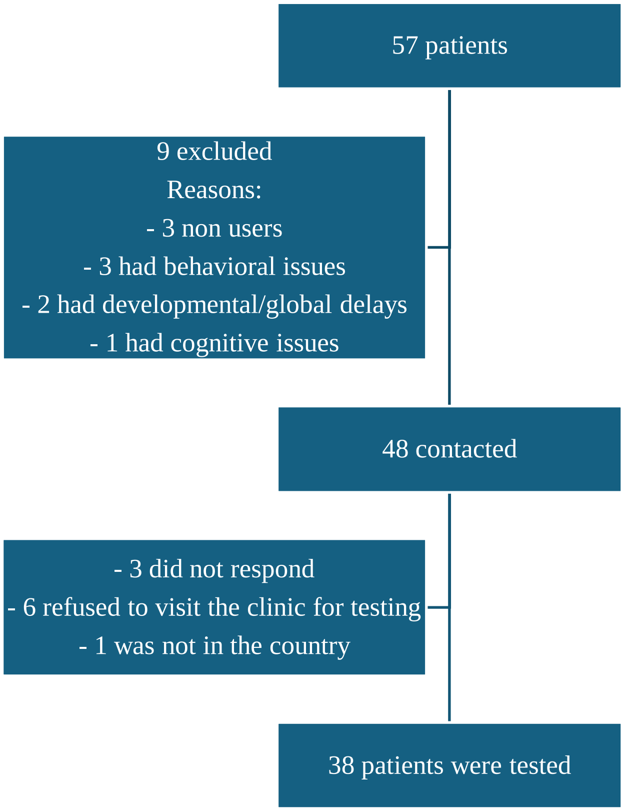

Figure S2. Set-up of the directional hearing assessment

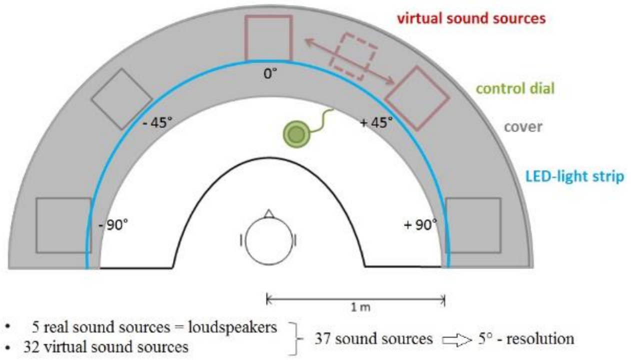

Figure S3. Differences in ERKI results with SCAN on between the different age groups: A. White noise; B. Pink noise; C. Speech in noise; D. Pulse noise

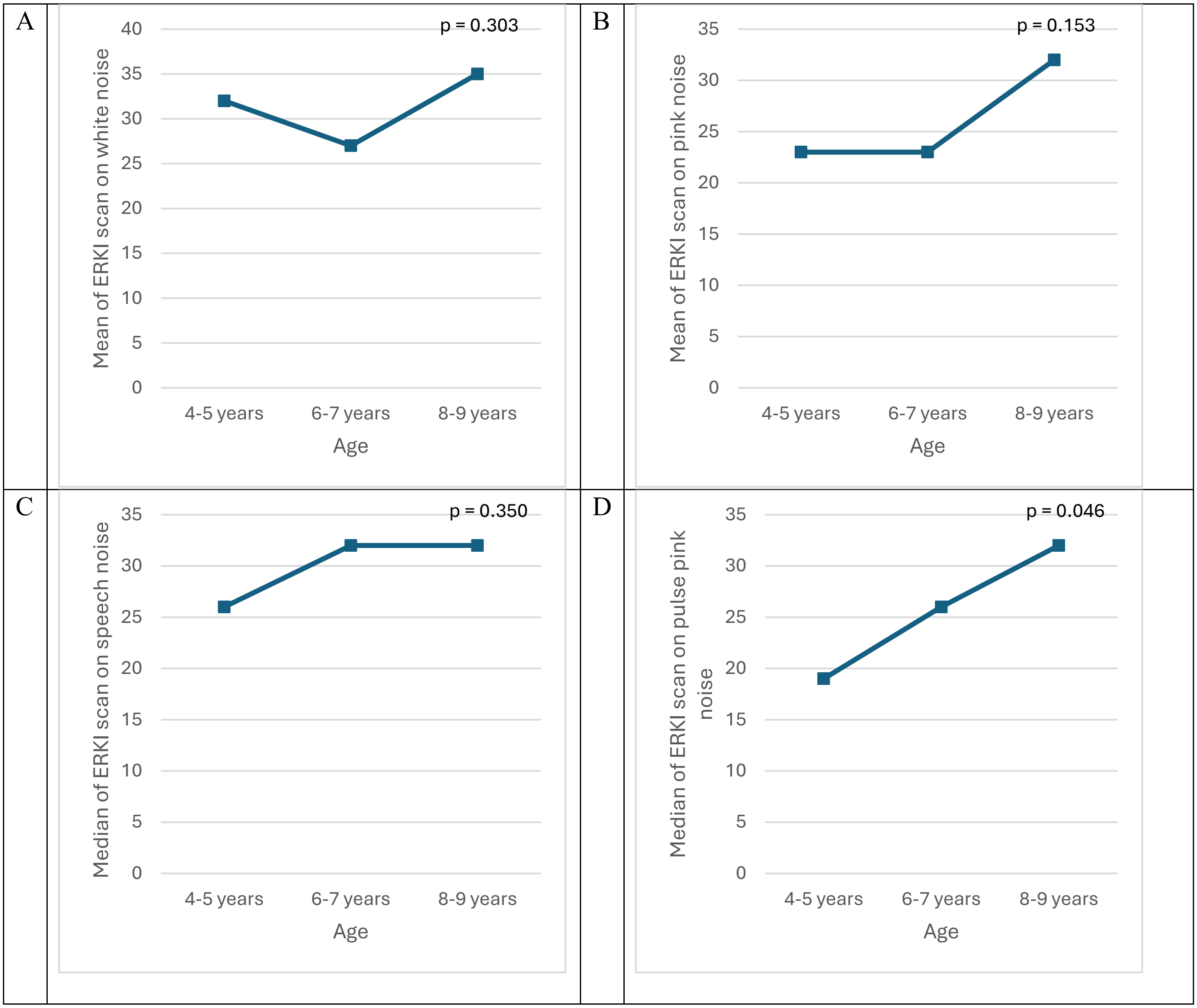

One Way ANOVA was used for normally distributed data and Kruskal Wallis for non-normally distributed data. Bonferroni pairwise comparison showed a significant difference in pulse noise results between the groups 4-5 years and 8-9 years.
